# Case Report: Management of a Multidrug-Resistant CMV-Strain in a Renal Transplant Recipient by High-Dose CMV-Specific Immunoglobulins, Modulation in Immunosuppression, and Induction of CMV-Specific Cellular Immunity

**DOI:** 10.3389/fimmu.2020.623178

**Published:** 2021-01-25

**Authors:** Vanessa Wiening, Tina Schmidt, Maximilian Dahmen, Sami Siam, Stefan Reuter, Hermann-Joseph Pavenstädt, Martina Sester, Barbara Suwelack

**Affiliations:** ^1^ Transplant Nephrology/Department of Internal Medicine D, University Hospital Münster, Westphalian Wilhelm’s University Münster, Münster, Germany; ^2^ Department of Transplant and Infection Immunology, Saarland University, Homburg, Germany

**Keywords:** multidrug-resistant cytomegalovirus, high dose immunoglobulins, renal transplant, case report, cytomegalovirus-specific cellular immunity

## Abstract

The management of multidrug-resistant strains of cytomegalovirus after solid organ transplantation is challenging. This case report demonstrates the successful treatment of a multidrug-resistant strain of cytomegalovirus that may represent a valuable option for problematic cases. This report illustrates the emergence of a multidrug-resistant cytomegalovirus (CMV) UL54 mutant strain in a renal transplant recipient with severe lymphopenia and thrombocytopenia. We show that the combined treatment with high-dose intravenous cytomegalovirus-specific immunoglobulins (CMV-IVIG) after the switch to a mammalian target of rapamycin (mTOR)-inhibitor and cyclosporine A was a successful treatment alternative to direct antiviral treatment with high-dose ganciclovir and foscarnet. This treatment was associated with a quantitative induction of CMV-specific CD4 and CD8 T cells that showed maturation in phenotype and functionality with decreasing viral load. Our case report illustrates that high-dose CMV-IVIG and conversion of immunosuppressive drugs to mTOR inhibitors and cyclosporine A can be a successful treatment in a situation where the use of direct antiviral drugs was considered insufficient.

## Introduction

Cytomegalovirus is one of the most common viruses causing infectious complications after kidney transplantation. Although the management of CMV has steadily improved in the last years due to improved diagnostics, increased knowledge of antiviral immunity, and the availability of specific antiviral drugs such as ganciclovir or its oral prodrug, CMV disease is still a major cause of post-transplant morbidity and mortality (1). A major therapeutic challenge arises when drug resistance develops not only to ganciclovir, but also to cidofovir or the nephrotoxic drug foscarnet (1). Although other antiviral treatment options such as letermovir and maribavir exist, both drugs are not yet approved for therapy in renal transplant recipients. Given the importance of the adaptive arm of the immune response including antibodies and functionally active effector T-cells for control of CMV replication, therapeutic substitution of CMV-specific immunoglobulins as well as induction of CMV-specific T cells may be important for a successful control of multidrug-resistant strains.

## Case Description

**Figure 1:**
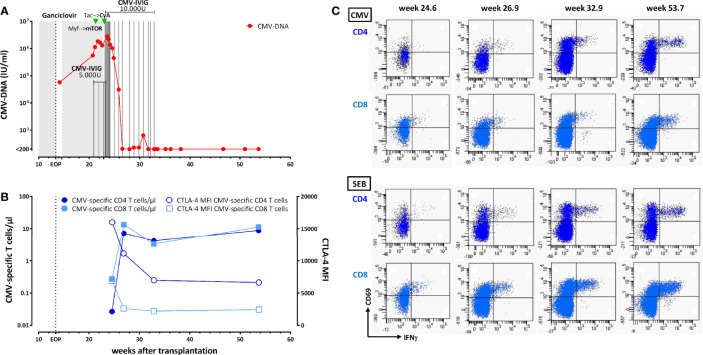
Control of cytomegalovirus (CMV) infection in a renal transplant recipient with a UL54 DNA polymerase gene mutation A834P. **(A)** Time course of CMV-DNAemia in relation to antiviral therapy with ganciclovir (i.v. and valganciclovir, shaded areas, for more information see text), modulation of immunosuppression from mycophenolate mofetil (myf) to an mTOR inhibitor and from tacrolimus (Tac) to cyclosporine A (CyA). In addition, the patient received CMV-specific i.v. immunoglobulins (IVIG). **(B)** Time course of absolute numbers of CMV-specific CD4 and CD8 T cells (black symbols) determined by flow-cytometry after specific stimulation with a CMV lysate and CMV peptides. In addition the expression level of CTLA-4 was determined [denoted as MFI (median fluorescence intensity)]. **(C)** Original data of CMV-specific CD4 and CD8 T cells determined over time (upper panels). The bottom panels show the time course in CD4 and CD8 T cells polyclonally stimulated with *Staphylococcus aureus* Enterotoxin B (SEB). Specifically activated T cells were characterized by expression of CD69 and interferon γ (IFNγ).

We report the course of a 51-years-old CMV IgG positive male recipient. He received his fifth kidney transplant from a CMV IgG positive deceased donor in January 2018. As he was highly immunized, the immunosuppressive drug regimen included ATG induction (Thymoglobuline^®^, Genzyme, 1.5mg/kg body weight on day 0, 2, 3, and 4 post transplantation), tacrolimus (Prograf^®^, Astellas, target level 6–8 ng/ml), mycophenolic acid (Myfortic^®^, Novartis, 720 mg b.i.d.) and prednisolone. Valganciclovir (Valcyte^®^, Roche) prophylaxis was given orally for 3 months. During the previous post-transplant periods, the patient had never developed any CMV-related complications. One week after stopping prophylaxis, asymptomatic CMV replication was detected at a level of 57,200 IU/ml (week 14, **Figure 1A**). Kidney function was stable with an eGFR CKD EPI of 63.2 ml/min/1.73 m², and laboratory parameters were within normal range.

Valganciclovir was initiated again at a therapeutic dosage adapted to renal function (Cockcroft-Gault formula). Despite antiviral therapy, viral load further increased. The patient was admitted to hospital, and antiviral treatment was changed from valganciclovir to i.v. ganciclovir (Cymeven^®^, Roche) from post transplant week 21 until week 23. By the time of admission, the patient had developed leukopenia (week 21), followed by thrombopenia (week 22), and malaise. He therefore received CMV-specific i.v. immunoglobulins (CMV-IVIG, Cytotect CP^®^, Biotest, 5.000 units per week, from week 21), and mycophenolate sodium was stopped and replaced by everolimus in week 22 (Certican^®^, Novartis, target level 3–5 ng/ml, mean trough level 4.69 ng/ml). As CMV viral load still increased, resistance testing was initiated (Institute of Virology, Ulm University Medical Center, Germany). The patient developed fever of up to 38.8°C and received antibiotic therapy with piperacillin-tazobactam (Tazobac^®^, Pfizer), although CRP levels never exceeded 2.5 mg/dl. Neither CT thorax scanning nor urine-testing nor blood cultures showed signs of infections. The patient developed diarrhea for three days. Clostridium difficile testing was negative and diarrhea ended spontaneously. In week 23, results from resistance testing returned and revealed the UL54 DNA polymerase gene mutation A834P (without concomitant mutation in the UL97 kinase gene). This mutation confers resistance against ganciclovir, foscarnet, and cidofovir (2). Although combined treatment with foscarnet and high dose ganciclovir is recommended in this situation (1), leukopenia and thrombopenia resulted in reluctance to follow this recommendation. As recent studies have shown a favorable effect of cyclosporine A and mTOR inhibitors on BK virus and CMV infection (3, 4), i.v. ganciclovir was stopped and immunosuppression was switched from tacrolimus to cyclosporine A (Sandimmun^®^, Novartis, target level 80-100 ng/ml) in week 23). In addition, the patient received high dose CMV-specific IVIG for 8 consecutive days (10.000 units per day, **Figure 1A**). Although viral load further increased to 2.813.000 IU/ml during week 24, this regimen resulted in an eventual decrease in CMV load for the first time later in week 24. The patient was discharged from hospital in week 25 with a viral load of 1.386.000 IU/ml, and monitoring was continued in an ambulatory setting. CMV-IVIG treatment was continued with 10.000 units once a week (**Figure 1A**). To analyze the patient´s cellular immunocompetence toward CMV, CMV-specific CD4, and CD8 T cells were quantified from whole blood after stimulation using flow-cytometry (5, 6). In brief, 450 µl of whole blood was stimulated with 32 µl/ml of a CMV lysate (Virion/Serion) and a mixture of 22 CMV-antigen derived MHC class I binding peptides [1 µg/ml, peptide sequences described in (7)] for a total of 6 h. After 2 h, 10 µg/ml brefeldin A (Sigma) was added to accumulate cytokines intracellularly. A non-infected control lysate (Virion/Serion) served as a negative control. CMV-specific cells were identified as described before (5, 6) after flow-cytometric staining as CD69 positive CD4 and CD8 T cells producing interferon γ (IFNγ). Moroever, induction of TNFα and IL-2 as well as expression of CTLA-4 was analyzed. A polyclonal stimulation with Staphylococcus aureus Enterotoxin B (SEB) was carried out to assess general immune responsiveness (5, 6). Interestingly, after discharge from hospital (week 25), CMV-specific CD4 and CD8 T cells were detectable (**Figures 1B, C**). However, consistent with high viral load, absolute counts/µl were very low, and their phenotypical and cytokine expression pattern showed typical signs of functional anergy characterized by high expression of CTLA-4 and limited ability to produce cytokines (**Figure 1B** and data not shown). By week 27, CMV viral load had decreased to 205 IU/ml. This was accompanied by a marked increase in CMV-specific T-cell numbers, which remained at a similarly high level when analyzed in week 33, at which time CMV-IVIG treatment was stopped. In week 34, the patient developed an acute humoral rejection, which was successfully treated with plasmapheresis, high dose corticosteroids and high dose intravenous immunoglobulins. CMV viral load remained low or below detection limit thereafter. One year after transplantation kidney function had stabilized, viral load was undetectable, and CMV-specific T-cell numbers had remained stable. Consistent with successful viral control, CTLA-4 expression and cytokine profile of CMV-specific T cells had normalized over time (**Figures 1B, C**).

## Discussion

CMV control by CMV-specific humoral and cellular immunity in kidney transplant recipients may be severely compromised by immunosuppressive therapy, which frequently necessitates specific treatment with antiviral drugs. However, drug resistance is an increasing problem in the management of CMV infection. The current international consensus guidelines on the management of cytomegalovirus in solid-organ transplantation recommend resistance testing in cases with persistent or recurrent CMV DNAemia or disease during prolonged antiviral therapy of more than six weeks (1). Our patient received a T-cell depleting induction treatment. As the depleting effect was shown to be particularly pronounced in CMV-seropositive patients on ATG treatment (8), this may have impaired CMV-specific cellular immunocompetence, thereby favoring uncontrolled replication after stopping prophylaxis (9). The management of the present case with the A834P mutation that conferred resistance to all currently recommended drugs posed a particular challenge. Based on severe leukopenia and thrombocytopenia, the recommended combined use of foscarnet and high dose ganciclovir (1) was not considered a relevant treatment option. This case report shows that high-dose CMV-IVIG may represent a valuable alternative in patients with multidrug-resistant strains, which has shown efficacy as adjunct therapy of CMV infections (10). In addition, although mTOR inhibitors may be more effective in CMV prevention than control (11, 12), early conversion of immunosuppressive drugs to a regimen containing an mTOR inhibitor may have at least in part contributed to viral control in our patient (11, 13). This may be related to its direct antiviral activity (4). In addition, mTOR inhibitor treatment in animal models was shown to specifically enhance pathogen-specific T-cell immunity, while maintaining suppressive activity on alloreactive T cells (14, 15). In humans, both *in vivo* and *in vitro* studies have shown that mTOR inhibitors contribute to an increase in specific cellular immune responses (16) with potent functionality (17, 18). This is in line with our observation that this regimen resulted in an increase in CMV-specific T cells as a sign of sufficient antiviral immunity that may have contributed to a decrease in viral load. Alternatively, the induction may be the result of homeostatic proliferation of CMV-specific T cells driven by viremia (19). The decrease in viral load may have also been supported by the neutralizing activities of CMV-specific IVIG. In addition, the opsonizing activity of immunoglobulins may favor uptake of CMV antigens into antigen presenting cells and thereby increase antigen-presentation and their stimulatory capacity toward CMV-specific T cells (20). Interestingly, induction of specific immunity was not only characterized by a quantitative increase in the number of CMV-specific CD4 and CD8 T cells but also to a maturation in phenotype and functionality that is similar to that of healthy CMV-positive individuals (21). We also have considered adoptive transfer of CMV-specific T cells as an alternative treatment option, but this was no longer necessary since the patient developed sufficient immunity on his own over time. Other experimental treatment options include letermovir, an inhibitor of the viral terminase complex UL56 that does not cross-react with valganciclovir, and maribavir, a selective orally bioavailable benzimidazole riboside. However, letermovir is not yet approved for therapy in renal transplant recipients (22). Given the absence of UL97 mutation, maribavir could have been an alternative in multidrug-resistant CMV infections, but only phase-2-trials for preemptive treatment of CMV infection exist so far (23).

A change in immunosuppression including combined treatment with CyA and mTOR inhibitor, as in this case may have been associated with a net reduction in the intensity of immunosuppression, which may have favored development of an acute humoral rejection episode. Interestingly, similar findings were recently reported in connection with a BK polyomavirus (BKPyV) infection, where the reduction of immunosuppression led to a concomitant increase in BKPyV-specific immunity and alloimmunity which was followed by a T-cell mediated rejection episode (TCMR) (24).

Our case report is limited by the fact that we did not analyze specific immunity directly at the time of stopping prophylaxis, which may have increased early vigilance for CMV-related complications. In addition, we did not determine antigenic specificities, which could be of additional value given the fact that low levels of IE-1 specific T cells were found to be highly predictive of subsequent viremia (9, 25). Moreover, the individual contribution of the therapeutic measures toward successful control of viremia cannot be assigned. Thus, we do not know, which part of therapy was successful or if it was the combination of all changes that eventually led to successful CMV control. Nevertheless, this case of a multidrug-resistant CMV strain with an UL54 DNA polymerase gene mutation A834P illustrates that high-dose CMV-IVIG and conversion of immunosuppressive drugs to mTOR inhibitors and cyclosporine A can be a successful treatment alternative to high-dose ganciclovir and foscarnet in CMV-seropositive patients with severe lymphopenia and thrombocytopenia, that may contribute to an induction of the patient´s own antiviral cellular immunity and long-term control of viral replication.

## Patient Perspective

This case report demonstrates the successful treatment of a strain of cytomegalovirus resistant to all commonly used antiviral drugs. The therapeutic options were therefore highly limited. The best therapy option was then applied in an interdisciplinary team under the supervision of clinical, virological, and immunological parameters. The treatment options were discussed in regular hospital internal conferences and telephone conferences with external experts. As a result, the immunosuppressive drugs were changed to substances that are known to be useful in viral infections. In addition, we applied CMV-specific hyperimmunoglobulins (CMV-IVIG) as adjunct therapy. Close monitoring of cellular immunity showed that CMV-specific T cells were largely absent during high-level viremia and recovered with improved control of viral replication.

## Data Availability Statement

The original contributions presented in the study are included in the article. Further inquiries can be directed to the corresponding author.

## Ethics Statement

Written informed consent was obtained from the individual(s) for the publication of any potentially identifiable images or data included in this article.

## Author Contributions

VW, MD, SS, SR, H-JP and BS treated the patient. TS and MS performed CMV-specific T cell analyses. All authors discussed the clinical case. VW, TS, MS, and BS wrote the manuscript. All authors contributed to the article and approved the submitted version.

## Conflict of Interest

The authors declare that the research was conducted in the absence of any commercial or financial relationships that could be construed as a potential conflict of interest.

## Abbreviations

µl, microliter; ATG, antithymocyte globulin; CD, cluster of differentiation; CKD, Chronic Kidney Disease; CMV, cytomegalovirus; CTLA-4, cytotoxic T-lymphocyte-associated protein 4; CyA, cyclosporine A; DNA, deoxyribonucleic acid; eGFR, estimated glomerular filtration rate; EPI, Epidemiology Collaboration; IFN γ, interferon γ; Ig, immunoglobuline; IgG, immunoglobuline g; IU, international units; i.v., intravenous; IVIG, intravenous immunoglobulins; kg,kilogram; m²,square meters; MFI, median fluorescence intensity; mg, milligram; min,minute; ml, mililiter; mTOR, mammalian Target of Rapamycin; myf, mycophenolate mofetil; SEB, Staphylococcus aureus Enterotoxin B; Tac, tacrolimus.
